# Impacts of Precipitation Events on Concentrations of Oxygenated Gas- and Particle-Phase Compounds Observed in the Amazon

**DOI:** 10.1021/acsestair.5c00163

**Published:** 2025-11-06

**Authors:** Sungwoo Kim, Gabriel Isaacman-VanWertz

**Affiliations:** Department of Civil and Environmental Engineering, Virginia Tech, Blacksburg, Virginia 24061, United States

**Keywords:** deposition, atmospheric chemistry, precipitation, atmospheric measurements

## Abstract

Removal of gases and particles by precipitation (wet deposition) is a critical process that significantly influences the transport and chemical transformation of atmospheric compounds. However, there are few studies that directly measure or constrain the rates of this process under real-world conditions. This work quantifies the net change in ambient concentrations during precipitation events (removal rates) of gas- and particle-phase organic compounds at a surface site near Manaus, Brazil, during the GoAmazon2014/5 campaign. Removal rates of identified and unknown compounds that have been previously classified into source-based clusters are measured during rain events and categorized based on estimated properties of compounds and clusters. Highly oxygenated gases, such as isoprene oxidation products, are removed during precipitation events with a median removal rate of 0.09 h^–1^ and the fastest analyte is removed at a rate of 0.22 h^–1^. Removal rates of particle-phase compounds are observed at roughly this median rate, while less soluble gases, such as terpenes, exhibit low removal rates. These results are roughly in agreement with prior theoretical estimates of wet deposition rates for comparable compounds, providing an empirical point of comparison while noting that our metric reflects the net influence of precipitation events rather than wet deposition alone.

## Graphical abstract

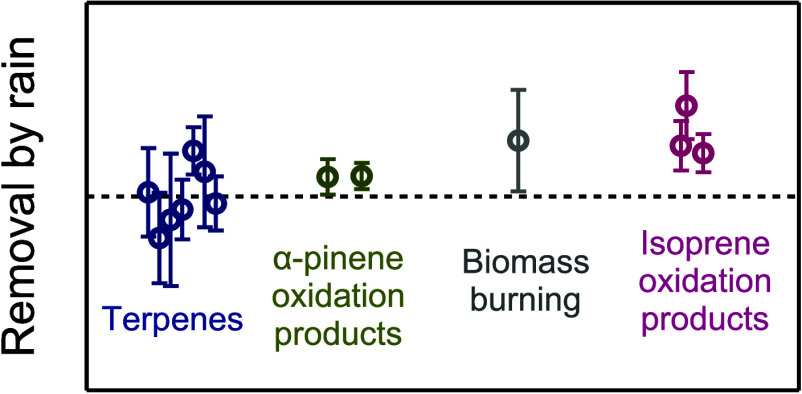

## 1 Introduction

The transport and chemical transformation of compounds in atmospheric samples depend highly on atmospheric conditions, including meteorological conditions, atmospheric oxidant concentrations, and inherent physicochemical properties of a compound. While atmospheric constituents can undergo photochemically driven oxidation reactions, forming particulate matter and/or producing ozone, they can also be removed through deposition to the Earth’s surface prior to or after reaction. Removal through the Earth’s surface can occur through dry deposition—loss to surfaces driven by turbulence and concentration gradients—or through wet deposition—scavenging by rainfall and clouds.^1,2^ In ecological and hydrological studies, wet deposition is expressed as a surface flux (μmol m^–2^ h^–1^) obtained from the product of rainfall amount and the compound concentration measured in bulk precipitation (*C*_rain_).^3^ This bulk-flux approach ties atmospheric budgets directly to the chemistry of the collected precipitation, yet it depends on compound-specific rainwater measurements. Atmospheric chemistry models more commonly express below-cloud scavenging as a first-order loss, and the scavenging coefficient Λ (s^–1^) is calculated by integrating the mass transfer contribution of all raindrop sizes, using parametrized raindrop size spectra and size-dependent collection efficiencies.^2^ The reciprocal of the scavenging coefficient then expresses the characteristic wet deposition time scale.

Though measurements of dry deposition have become more common through fast measurements of deposition and emission fluxes,^4^ relatively few observational constraints exist for wet deposition, and the relative importance of these processes is not well-known. Observational constraints for wet deposition are primarily available for particles, for which conservative tracers (e.g., black carbon and other inorganic species) can be collected during precipitation events and compared to modeled processes.^5–7^ In models, wet deposition significantly outcompetes dry deposition as a loss process for sulfate- and organic-rich particles, and both occur on the time scales of days to weeks.^8,9^ In contrast, model deposition of gases suggests dry deposition may occur on time scales of hours in some environments, outcompeting wet deposition, which typically takes days due to the sparsity of rain events.^10^ Many of the oxygenated organic compounds observed in the atmosphere do not have known identification or may be transformed by uptake into collected water; therefore, it is difficult to relate rainwater concentrations to atmospheric concentrations for these types of compounds, stymying the typical approach to wet deposition measurements. Consequently, only limited observations exist to benchmark model predictions of wet deposition of oxygenated gases, and the global variability of time scales for these processes is not yet well-studied.

Wet deposition operates through two primary mechanisms: in-cloud and below-cloud scavenging. In saturated air, particles act as nuclei for water vapor to condense upon, forming cloud droplets. These droplets eventually precipitate and are removed from the atmosphere. For below-cloud scavenging by raindrops, the efficiency depends on factors such as rainfall intensity, droplet size distribution, and properties of the species being scavenged. For particles, the theoretically relevant property is primarily size distribution, as it governs collision probability. However, field measurements indicate that observed scavenging rates are one to two orders of magnitude faster and exhibit weaker size dependence than theoretical predictions, likely due to turbulent transport and mixing, cloud microphysics, and aerosol microphysics.^11,12^ Consequently, most models use a parametrization for this process, and some models estimate that both in-cloud and below-cloud scavenging are approximately equally important in the removal of water-soluble particles.^7^

For gases, the most relevant property for wet deposition is affinity for water (i.e., Henry’s Law Constant, approximately “solubility”) as it governs dissolution into the raindrops. Gases with high water solubility are taken up by droplets irreversibly at the collision limit, while less soluble compounds reach equilibrium with raindrops and are lost less efficiently. Prior work has estimated that above a certain threshold for Henry’s law (∼10^5^ M/atm at 298 K), removal by wet deposition is no longer limited by solubility and plateaus at a maximum removal rate governed by collision frequency and thus rainfall characteristics.^10^

Dry and wet deposition of oxygenated gases has been modeled to significantly impact downstream aerosol formation and atmospheric composition.^13,14^ While dry deposition generally is modeled to dominate over wet deposition as the larger global sink, implementation of deposition processes in models remains relatively uncertain and lacks observational constraints, particularly for wet deposition. Most existing estimates of removal rates rely on theoretical relationships rather than direct measurements,^15–17^ as there are very few published observational constraints for below-cloud scavenging of oxygenated organic gases. Observed decreases in particle concentrations during precipitation exceed rates estimated by commonly used below-cloud scavenging parametrizations,^11^ so it is critical to validate current estimates with observations in order to improve the representation of these processes in atmospheric models. Changes in the concentrations during precipitation have been observed for a wide range of less-soluble gases. Of particular relevance to this work are observations of greenhouse gases and biogenic emissions of hydrocarbons in the Amazon, which highlight the complex impacts of precipitation, causing shifts in air masses (i.e., convective downdrafts and gust fronts from rain-cooled outflow) and modifying biological and ecological processes.^18,19^ However, few measurements are available of highly oxygenated gases that may partition to the particle phase or react efficiently to form aerosol, and such data are valuable even in the context of these complexities.

In this work, instead of deriving Λ from measuring or parametrizing rain characteristics, we quantify the net fractional change in near-surface concentration during each rain hour. This concentration-change method provides event-scale removal coefficients (R, h^–1^) that directly reflect real-world processes (i.e., including below-cloud scavenging, dry deposition, vertical mixing, and emission perturbations) that act on the time scale of our observations. This approach has been examined in detail for measuring the removal of particles as a function of size and rainfall rate,^11^ but has not been previously applied to condensable oxygenated gases because few in situ measurements exist for these compounds. However, given the importance deposition of such gases has for aerosol formation and atmospheric composition,^10,13^ observational constraints on the process are critical. In a previous work,^20^ we have demonstrated that ambient atmospheric samples can be systematically processed to detect unique analytes and generate their corresponding time series, which provides a rich data set for directly observing impacts of precipitation on concentrations of a wide range of compounds. This data set includes a wide range of compounds, such as terpenes, alkanes, and various isoprene oxidation products, as well as a significant number of compounds that could not be identified due to the lack of matching spectra in the NIST/NIH/EPA mass spectral databases.^21^ These time series form the platform for all subsequent removal rate analyses presented here. More details about the sample data are described in Section 2.1, and the treatment of unidentified compounds is detailed in Section 2.2.

In this study, we aim to (1) quantify the changes in concentrations of compounds to quantify removal rates during precipitation and (2) interpret these removal rates within the contexts of their identity when available, their source-based clusters, and physicochemical properties.

## 2 Materials and Methods

### 2.1 Sample Data Sets

The data set used in this work consists of 408 analyte time series of gas- and particle-phase compounds collected from ambient air. Raw data were collected using a semi-volatile thermal desorption aerosol gas chromatograph (SV-TAG) with in situ derivatization. The instrument setup utilized two identical cells operating in parallel, with one cell optionally coupled to a denuder to exclusively capture particle-phase signals. In this configuration, total (gas plus particle phases) signals were collected hourly, while particle-phase-only signal was collected every other hour on one of the cells. Hours in which both cells captured total signals were used to correct for any channel-dependent differences. In hours in which the denuder was employed upstream of one cell, the fraction of each compound in the particle phase was calculated by comparison between cells, providing a gas–particle partitioning metric that is later used to interpret dominant removal mechanisms. Gas-phase concentrations were then derived by difference. For each hour in which a denuded (particle-only) measurement was available, the sensitivity-corrected particle-phase concentration was subtracted from the coincident total (gas + particle) concentration measured on the parallel cell. Of these compounds, 15 analytes are predominantly gaseous (<10% particle fraction) and 165 are predominantly particle-phase (>90% particle fraction). This particle-phase set includes both truly particle-phase analytes and internal standards/contaminants that identically appear on both cells and thus have a high apparent particle fraction. The remainder exhibit intermediate partitioning. External standards were injected into both cells approximately every 7 h for calibration.^22^ Signals from external standards were not included in the analysis of data sets as they do not reflect real-world changes in analyte concentrations. A more detailed description of sampling is provided by Isaacman-VanWertz and coauthors.^23^ Due to the high inherent temporal variability in precipitation, quantitative removal rates are calculated only for hourly (gas plus particle) data, with fraction in the particle phase used primarily for data interpretation.

The data set used here was collected during the Intensive Operational Period 1 (IOP1) of the GoAmazon 2014/5 campaign in Manacapuru, Brazil. The site is ∼70 km downwind of Manaus, Brazil, and data were collected during February and March of 2014. Calibrated time series for analytes with definitive identifications are publicly available through the Department of Energy Atmospheric Radiation Monitoring (DOE ARM) data archive, which can be found at (https://iop.archive.arm.gov/arm-iop/2014/mao/goamazon/T3/goldstein-svtag/). In addition, temperature, relative humidity, and precipitation were measured at the site using a Vaisala WXT520 weather transmitter, which employs a piezoelectric sensor for precipitation measurement as part of the Aerosol Observing System Meteorology (AOSMET) data set.^24^ The data was recorded at a temporal resolution of 1 s, with a measurement error range of 5%. These data are publicly available through the DOE ARM database.^25^ To facilitate effective comparison with the time series of analyte concentrations, the 1 s precipitation data was summed into hourly average precipitation rate (Figure S1), resulting in a data set expressed in millimeters per hour. A large fraction of rain events in the Amazon lasts for less than an hour,^18^ but the hourly time resolution of the measured organic compounds necessitates this averaging.

### 2.2 Clustering and Analysis of Unidentified Compounds

In previous work, this data set was used to characterize unidentified compounds by clustering them with the identified compounds based on similarities in their temporal variations, resulting in 8 distinct clusters, each representing different chemical structures or origins. Details on clustering can be found in Kim et al.^20^ In this work, clusters are primarily used for the interpretation of the unidentified analytes, increasing statistical power by allowing conclusions to be generalized to related compounds, not just a subset of identified compounds. The instrument setup described in Section 2.1 was capable of simultaneously measuring both gas- and particle-phase signals of oxygenated organic compounds.^22^ Thus, for each analyte, the particle fraction (i.e., the average ratio of the particle-phase concentration to the gas plus particle-phase concentration) was calculated. Since below-cloud scavenging proceeds mainly by inertial/impaction capture for particles and by dissolution for gases, an analyte’s particle fraction helps indicate which of these two processes—particle impaction/interception or gas dissolution—is likely to dominate its removal. By comparison of the concentration time series of these analytes with precipitation data collected at the sampling site during the same period, the removal rate of each analyte was estimated as a measure of the net concentration change during precipitation events for these oxygenated compounds. It should be noted that these changes in concentration may also include effects from transport of other air masses or other effects, therefore representing an overall effect of precipitation that can be used to inform the understanding of wet deposition within that context. For the compounds for which no definitive identification is possible due to their absence in libraries, removal rates were interpreted in the context of the clusters into which they are grouped, and physicochemical parameters were estimated from mass spectra.

### 2.3 Estimation of Removal Rate during Precipitation

The removal rate per hour of analyte during a rain event is quantified as the fractional change in concentration after and during the hour in which precipitation occurred:

$$\mathrm{scavenging}\;c\mathrm{oefficient},\Lambda \approx \mathrm{removal}\;\mathrm{rate}\;[h^{-1}]=\frac{C_{0}-C_{t}}{\max(C_{0},C_{t})}\times \frac{1}{\Delta t}$$ 1

where *C*_0_ is the concentration of an analyte recorded at the start of the hourly sampling interval that contains precipitation (i.e., the representative concentration for that rain-affected hour), and *C_t_* represents the concentration of the analyte after *t* time, during which precipitation occurs. The denominator uses max (*C*_0_, *C_t_*) so that the fractional change is normalized to the larger of the two concentrations. Due to the hourly time resolution of the concentration measurements, Δ*t* is typically taken as 1 h, and precipitation is assumed to occur throughout the hour. This assumption treats wet deposition as a process that is linear with precipitation rate, which is true of the governing equations; if rainfall occurs only during the fraction of the hour sampled for *C*_0_, the calculated coefficient would be reduced due to a reduced starting concentration, and thus, this assumption is conservative. If the concentration data in the subsequent sample (i.e., after 1 h) is unavailable (due to, e.g., calibration or other instrument issues, the next available concentration data is used as *C_t_*, with the appropriate Δ*t*. Resulting values range from −1 to 1, representing the limits in which the analyte was not present prior to the rain event and then increased (−1), and the analyte was not present after the rain event (1). This removal rate is related to, but not identical with, the classical wet deposition scavenging coefficient, Λ. Mathematically, Λ is the first-order loss constant in an exponential decay, whereas our metric is the fractional concentration change. Because the observed relative change in concentrations is typically relatively small, in the range of −0.2 to 0.2, a linear approximation of the exponential decay does not introduce significant error. The observed removal rate reflects not only below-cloud scavenging but also any simultaneous vertical mixing (e.g., convective downdrafts) or short-term emission shifts that may accompany rainfall. Throughout the paper, we therefore retain the term removal rate to emphasize this combined influence, noting that under conditions where wet deposition dominates, it can be interpreted as an effective first-order scavenging coefficient but may include influence from other processes that cannot be deconvolved.

The sample collection period spanned February 14 to March 25, 2014. Over a total of 931 h, precipitation was observed for 179 h, with hourly intensities ranging from 0.01 to 29.63 mm/h. Given that the data were measured with a resolution of 1 s and the minimum detectable amount was 0.01 mm, a recorded precipitation rate of 0.01 mm/h indicates rainfall occurred for only 1 s within the respective hour. From the total rainfall intensity range of 0.01–30 mm/h, the number of hours with recorded precipitation as a function of rain intensity was plotted to examine general trends, revealing an exponential decline in frequency as intensity increased (Figure 1). Although the 0.01–1 mm/h intensity bracket recorded the most rainfall hours (106 h), the average duration of rainfall within this bracket was just 26 s. The samples collected in this study were taken hourly and represented the concentration of analytes at each hour, so short-duration (and low-intensity) rain events are unlikely to yield measurable changes in hourly analyte concentrations. Consequently, only hours with precipitation exceeding 1 mm/h were considered precipitation events for analysis in this study. Although 73 h initially met this criterion, after excluding standard runs and missing data, 43 rain events remained for analysis, focusing on periods with significant rainfall while retaining a substantial portion of the rain-affected data points. For the 43 h with intensities greater than 1 mm/h, mean rain duration rises from roughly 2 min in the 1–2 mm/h bin to about 21 min for events above 10 mm/h (Table S2). Since even the longest bursts occupy well under half of the 60 min sampling window, the hourly averaging period captures the full rain event while still leaving sufficient pre- and postrain time to register concentration changes, making the 1 h window a suitable basis for the removal-rate calculation. Because the calculation is based on a concentration ratio, the value we report represents the net fractional change during each rain hour rather than an isolated wet-scavenging coefficient. Hourly removal, therefore, reflects the combined influence of below-cloud scavenging and any co-occurring processes (e.g., dry deposition, biological processes, re-evaporation, or rain-induced mixing). The relative importance of those additional terms is evaluated further in Section 3.1.

**Figure 1:**
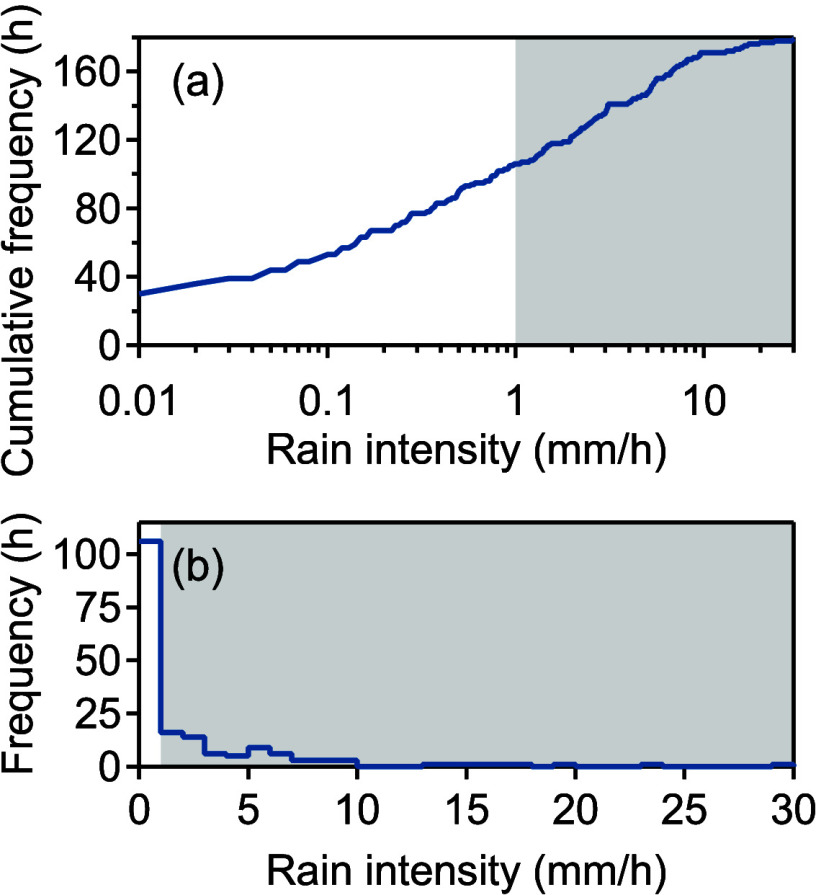
Histograms of hourly rainfall intensity, with frequency represented by the total number of hours for each intensity range. (a) Cumulative shown in log space and (b) noncumulative shown in linear space. Approximately than 40% of wet hours occur at intensities above 1 mm/h (shaded region); fewer than 5% exceed 10 mm/h, and the single highest hour reaches 29.6 mm/h.

Prior work has formally and empirically demonstrated that the precision for each concentration measurement using this instrument is <15%, while the ratio of two measurements, as used for the calculated removal rate, has an uncertainty of 15%.^22^ This represents the uncertainty in an individual data point. If only instrumental error is considered, propagating that 15% through the 43 rain events in this study yields a standard error (SE) on the median removal rate of roughly ±2–3% of the median (details in Supporting Information, S5). In practice, however, the empirical SE for each compound’s median removal rate is broader because it includes real atmospheric variability in addition to instrument error, and these compound-specific values for 74 identified compounds are reported in the SE column of Table S1 and shown as error bars in Figure 4.

For all 408 analytes, removal rates were calculated for the entire sampling period, and the median value of all rain events is taken as the representative removal rate for the specific analyte. This data set includes 74 analytes for which a definitive identification is available through matching to mass spectral libraries and/or authentic standards. These analytes include internal standards introduced during data collection, compounds used in external standards for calibration that may or may not also be present in atmospheric samples, and analytes representing ambient atmospheric compounds for which a mass spectrum is known and are listed in Table S1. For all other analytes, no identification was possible, and analysis is limited to estimation of physicochemical properties using the Ch3MS-RF machine-learning-based model for property prediction^26^ and by interpretation using the cluster to which the compound has been assigned. In a detailed examination of the approach used in this work,^11^ it has been shown that natural atmospheric variability leads to significant scatter in observed changes in concentration, so central tendencies of a larger number of data sets provide a more statistically meaningful estimate of removal rates. For this reason, most removal rates in this work are provided as medians of all rain events for individual analytes, and medians of clusters or groups of compounds provide further statistical robustness for interpreting general trends in processes for related or chemically similar compounds.

## 3 Results and Discussion

### 3.1 Removal of Individual Analytes

Removal rates for the 74 identified analytes ranged from −0.06 to 0.14 h^–1^. To evaluate potential biases in the calculation approach, the apparent removal rate was calculated for an internal standard introduced on every sample (perdeuterated hexadecane, *d*-C_16_). The signal of this compound is used to track instrument sensitivity and is independent of ambient conditions, so it is expected to remain unaffected by precipitation. As expected, precipitation has no effect on run-to-run instrument variability (Figure 2), with calculated removal rates low and centered around 0 (median value of 0.01 h^–1^). Furthermore, variability is dominated by long-term trends in instrument operation, and the run-to-run change in signal of this compound is the same for samples impacted by precipitation (red circles) as for those without precipitation (gray circles). This supports removal rates measured for other compounds, indicating that they do not represent artifacts due to the calculation or other aspects of this approach.

**Figure 2:**
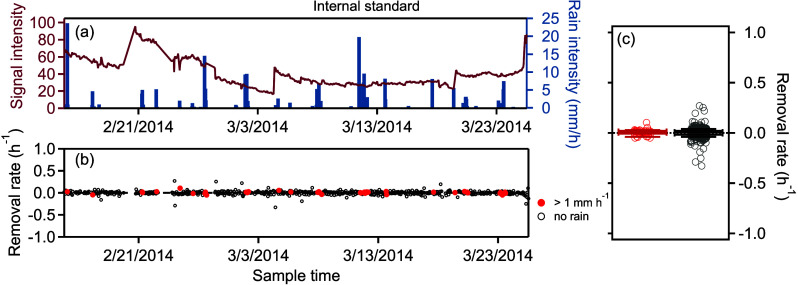
Calculated removal rates of an internal standard. (a) Signal over time of perdeuterated hexadecane, an internal standard introduced to every sample (purple line) alongside the precipitation rate (blue bars). (b) Calculated removal rates of concentrations during precipitation events (red circles) and during nonrainfall periods (gray circles). (c) Box-and-whisker plots of the 43 rain events and 514 nonrain hours, with shaded band indicating ± 1 standard error of each median.

Conversely, the removal rate of a known isoprene oxidation product, trans-2-methyl-1,3,4-trihydroxy-1-butene (C_5_H_10_O_3_, commonly referred to as a C_5_-alkene triol, though this identification may not be accurate^27^), during precipitation events exhibits a broader distribution skewed toward positive values, ranging from −0.36 to 0.85 h^–1^, with a median of 0.14 h^–1^ ± 0.05 (Figure 3). Though overall trends in the signal are dominated by a clear diurnal trend, this value represents a general tendency for concentrations to decrease during precipitation events. Recent work^28^ has suggested that the instrument signal from the C_5_-alkene triols may in part represent particle-phase uptake of isoprene epoxydiol and that some previously recognized compounds in this family may actually be cyclic tetrahydrofurans, while other isoprene oxidation products may derive in part from instrument-driven transformations.^29^ For the present work, we make no particular claim as to the structure of these compounds, which are uniquely identified by their chromatographic retention time and mass spectrum (in this case, quantification ion *m*/*z* 231), but rather use them as previously identified tracers for isoprene oxidation that provide insight into oxygenated gases and biogenic oxidation products. Although rainfall can trigger rapid biogenic emissions of isoprene and changes to the biological environment, such as canopy cooling, reduced sunlight, stomatal closure, microbial activity, and mechanical disturbance by raindrops,^18^ these changes occur over minutes to roughly 1 h and therefore are unlikely to influence the isoprene oxidation product examined here, whose formation requires a few hours of atmospheric processing. A rise and fall of the precursor during precipitation may therefore change the concentration downstream, but should not be correlated with the contemporaneous precipitation rate. By contrast, C_5_-alkene triol exhibits a consistent concentration decrease that aligns with the rain events. Therefore, the observed removal rate can be interpreted as reflecting precipitation-associated processes (wet scavenging and/or any concurrent air mass shifts) rather than random variability in precursor concentration.

**Figure 3:**
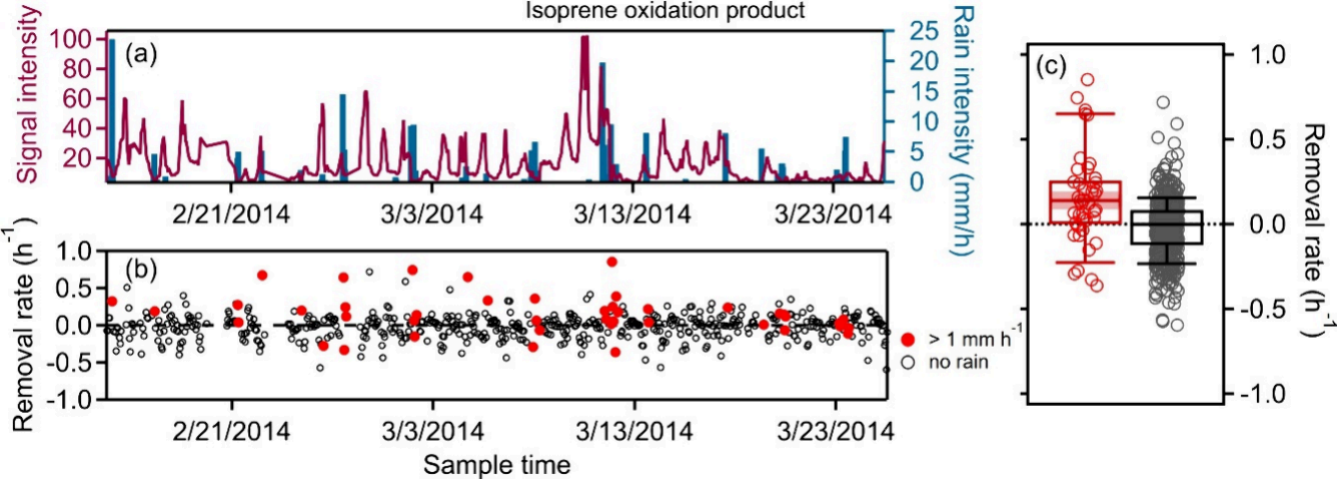
Calculated removal rates of a highly oxygenated isoprene oxidation product. (a) Signal over time of C_5_-alkene triol (purple line) alongside the precipitation rate (blue bars). (b) Calculated removal rates of concentrations during precipitation events (red circles) and during nonrainfall periods (gray circles). (c) Box-and-whisker plots of the 43 rain events and 514 nonrain hours, with shaded band indicating ± 1 standard error of each median.

The effective removal rate of the isoprene oxidation product during nonprecipitation periods (i.e., fractional change in signal as calculated in eq 1, gray circles in Figure 3b) generally exhibits smaller sample-to-sample variability and no bias toward reductions in concentration (median of <0.01 h^–1^), reinforcing that the systematic decrease occurs specifically during rain. Since internal standard signals vary only with instrumental precision, their fractional changes during nonrain hours span a narrow range (Figure 2b), whereas the C_5_-alkene triol concentration is influenced by atmospheric variability such as emissions, photochemical production, and boundary-layer mixing; this additional variability explains the wider scatter of gray points in Figure 3b. The side-by-side box-and-whisker comparison in Figure 3c shows that during precipitation, although there is scatter for individual points, there is a clear shift toward net removal of this compound that is not observed on average when it is not raining. The observed removal during precipitation is statistically significantly different from changes in concentration during nonprecipitation at the level of *p* < 0.001 (Wilcoxon rank-sum test), indicating that the reduction of this oxygenated gas during precipitation is a robust result despite some inherent variability in the quantitative rate.

The effects of wet scavenging and co-occurring processes, such as shifting air masses due to convective downdrafts, cannot be separated in the observed changes in concentration. To examine the potential importance of the latter process, shifts in the ozone concentrations can provide some insight into the prevalence of convective downdrafts. Convective downdrafts can episodically elevate nighttime ozone concentration in the central Amazon by several ppb.^30^ Average profiles of ozone for the 2 h period before and after the onset of each nighttime rain (20:00–05:00 local time) do not exhibit clear enhancements of ozone during precipitation events (Figure S7), suggesting that there is no clear evidence for convective downdrafts driving the observed shifts in concentrations. However, there are precipitation events that are often associated with increases in windspeed (Figure S2), so some changes in air mass are likely occurring and contributing to observed impacts of precipitation.

Perdeuterated hexadecane (Figure 2) and C_5_-alkene triol (Figure 3) represent roughly the two extremes of calculated removal rates for identified analytes. The median values for a subset of identified compounds that are known to be internal standards, resolved terpenes, α-pinene oxidation products, biomass burning tracer, and isoprene oxidation tracers are 0.01, −0.00, 0.03, 0.09, and 0.08 h^–1^, respectively (Figure 4). As expected, removal rates for internal standards are tightly clustered around and typically within an uncertainty of zero, providing confidence that the analysis approach does not produce artificial or apparent removal rates where none should be expected. Resolved terpenes include sesquiterpenes (C_15_H_24_) and diterpenes (C_20_H_32_), some of which have known identities, and some of which exhibit clear mass spectral fragmentation patterns of these compound classes but could not be definitively identified due to similarities between spectra. These terpenes exhibit high variability in their median removal rates, ranging from −0.06 to 0.07 h^–1^. In general, the central tendency of this class is neither to be removed nor increased during precipitation, as the median of these compounds is approximately zero. However, the high variability of these compounds relative to internal standards demonstrates the natural variability in real-world concentrations and may arise from a combination of precipitation and other meteorological or biological factors (such as vertical mixing, light and temperature dependence, and soil moisture)^31^ that modulate emissions and mixing on hourly time scales. Measurements of other terpenoids during precipitation events have observed complex temporal dynamics, most notably increases in concentrations of monoterpenes during peak precipitation attributed to enhanced emission from wetted surfaces.^18,19^ Similarly, decreases in concentrations attributed to entrainment of air from above during convective rain events have been observed in the Amazon,^18^ though in contrast to other studies, we have not observed precipitation-driven changes in nighttime ozone concentrations. These opposing influences (e.g., surface re-emission, mechanical sweep out of particle-phase, adsorption or wash-off from foliage, and sporadic entrainment from cleaner air) likely contribute to the high variability and, in some cases, apparent increases of terpenes during events. Overall, though, gas-phase scavenging of hydrocarbons such as these is not expected to be efficient, and the near-zero median we observe is interpreted as the net outcome of several competing processes rather than evidence of strong solubility-driven washout. Furthermore, a shift in air masses would be expected to drive large changes in concentration due to the strong concentration gradient for emitted terpenoids,^18^ so a median value of zero for these hydrocarbons suggests this process is not dominating the observed changes in concentration. Beyond vertical mixing, two additional mechanisms can further modulate terpene concentration change during rain: short-lived soil- and litter-emission bursts triggered by wetting and enhanced dissolution in rainwater with dissolved organic matter as a cosolvent. Convective showers in the central Amazon have been shown to release biogenic VOCs from damp soils, bark, and leaf litter.^18,19^ These sources can offset scavenging losses, especially for weakly soluble terpenes. Laboratory studies^17^ demonstrate that humic acids and other similar dissolved organic substances can increase the effective solubility of hydrophobic VOCs, providing an extra rain-induced removal beyond gas–water partitioning. The interplay of these opposing influences may contribute to the higher variability (−0.06 to +0.07 h^–1^) and the near-zero cluster median seen here. Consequently, we cannot rule out a modest compensatory influence on the terpene cluster.

**Figure 4:**
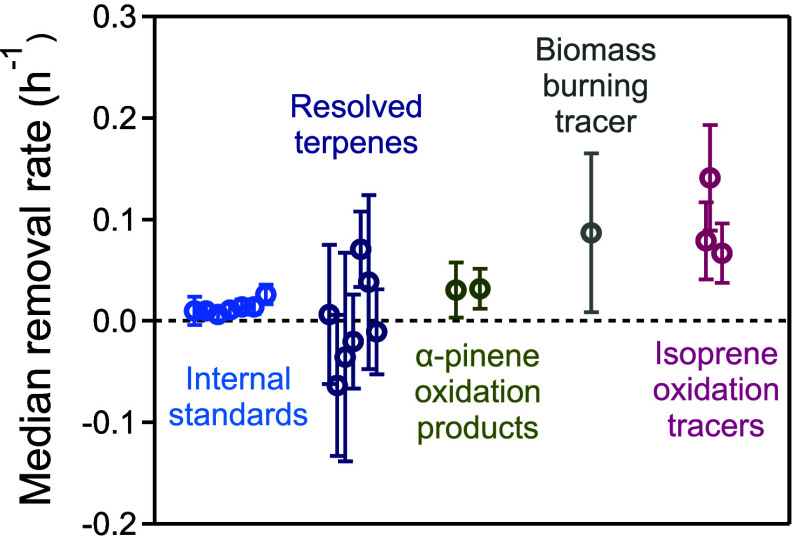
Removal rates for a subset of identified compounds include internal standards, resolved terpenes, levoglucosan (a biomass burning tracer), and oxidation of α-pinene and isoprene. For each compound, the symbol marks the median removal rate across all rain events, and the vertical error bar represents ±1 standard error (SE), calculated from the empirical standard deviation of that compound’s removal rates across all precipitation events.

Both identified α-pinene oxidation products (pinonic and pinic acids) are removed at a median rate of 0.03 ± 0.02 h^–1^ (∼3% per hour), statistically nonzero, but somewhat slower than previously estimated values for highly soluble gases.^10^ Both compounds are primarily in the gas phase, and have moderate to high Henry’s law constants above the threshold for maximum deposition (roughly 10^6^ and 10^9^ M/atm for pinonic and pinic acids, respectively),^23^ so would be expected to be removed fairly efficiently. In addition, submicrometer aerosol in the Amazon is typically highly acidic,^32^ and rainwater is typically also acidic.^33^ At lower pH values, only a fraction of acids dissociate in the particulate aqueous phase, lowering the effective solubility well below the bulk values of 10^6^–10^9^ M/atm. Bi and Isaacman-VanWertz^10^ used a Monte Carlo simulation model to follow the decay of a unit gas mass until 1/e of the original mass is reached. For highly soluble gases (H ≥ 10^5^ M/atm), the resulting wet deposition time scale is 4–6 h, providing a collision-limited benchmark for comparison with our observations. This modeled rate (4–6 h time scale) is somewhat faster than observed rates (∼0.03 h^–1^), but of a similar scale, and agrees with the fastest removal rates observed for individual compounds in this work. This discrepancy may be due to uncertainty in Henry’s law constants, reduced solubility due to particle acidity, or the impact of the particle-phase fraction. These compounds are primarily in the gas phase but have some particle-phase component, so the removal rate is a combination of wet scavenging of gases and particles, which occur through different processes. The impact of particle scavenging can be explored in part through the biomass burning tracer, levoglucosan, which is almost entirely in the particle phase. Levoglucosan is highly water-soluble, strongly hygroscopic, and is carried predominantly in submicrometer smoke particles. The estimated removal rate of levoglucosan was approximately 0.09 ± 0.08 h^–1^, which is notably higher than the median values observed in the first three groups and therefore serves as a practical benchmark for particle-phase removal, though high variability in the changes in concentration of this compound yields high uncertainty. Laakso et al.^11^ used a similar methodology (real-time measurements during rain events, but for size-resolved particle counts at a faster time resolution) to estimate the below-cloud wet scavenging coefficient for particles in the size range sampled here (submicrometer) for the rain intensities studied here (greater than 1 mm/h) to range from 0.036 to 0.18 h^–1^. A particle removal rate of roughly 10% per hour is consequently in line with the expected range and approaches the expected time scale for removal of highly soluble gases, both in terms of previous models^10^ and observations for the efficiently removed gases in the present study. Taken together, these results indicate that scavenging of particle-phase compounds—illustrated here by levoglucosan and consistent with literature ranges—occurs at expected magnitudes. Therefore, particle-phase removal alone is unlikely to entirely explain the lower-than-expected rates noted for some gas-phase species. The reason for lower-than-expected removal rates for pinic and pinonic acids, therefore, remains unclear but may indicate inaccuracy in the prediction of Henry’s Law constants or impacts of acidity.

The most highly oxygenated gases identified within this data set are the isoprene oxidation products, which include two 2-methyltetrols (2-methylthreitol and 2-methylerythritol) and the C_5_-alkene triol shown in Figure 3; though a second compound in the C_5_-alkene triol/C_5_H_10_O_3_ family was measured, the signal was too low to provide a reliable calculation of removal rates. The median removal rates of these compounds range from 0.07 to 0.14 h^–1^. These isoprene oxidation tracers are highly oxygenated and have high Henry’s law coefficients (10^6^–10^12^ M/atm for 2-methyltetrols),^23,34^ and are therefore expected to be removed at approximately the maximum wet deposition rate. The observed rates approach the estimated 4–6 h wet deposition time scale of highly soluble gaseous organic compounds^10^ (28 to 64% removal after 4 h, i.e., depletion to roughly 1/e or one lifetime). The removal rate observed here for oxygenated gases is comparable to that previously reported for nitric acid. Henry’s law constant of nitric acid lies near the bottom of this plateau for which maximum removal is expected (≈10^5^ M/atm). Reported below-cloud scavenging time scales range from 1 to 20 h for rainfall rates of 1–20 mm/h,^35,36^ and rainwater budget analyses give a ∼9 h time scale at 1 mm/h.^37^

The general agreement between the observed removal rates of the most soluble identified gases, approximately, published scavenging rates of nitric acid, and the previously modeled deposition rates supports the important conclusion that, as a community, our understanding of the processes driving removal of oxygenated organic gases by below-cloud scavenging reasonably describes real-world conditions. These observations cannot be explained by dry deposition. Field studies over forest canopies report daytime dry-deposition velocities for highly oxygenated isoprene products of 0.5 cm/s, with upper limits around 2.5 cm/s (leading to removal rates of roughly 6% per hour).^38,39^ Since canopy wetting and stomatal closure during rainfall reduce deposition velocity further,^40^ and dry deposition is also expected to be observed across all nonrain events, dry deposition during rainfall is somewhat too slow to explain the ∼9% h^–1^ median observed decrease in these compounds and would not be expected to be preferentially observed during precipitation.

### 3.2 Removal of Unidentified Analytes

While identified compounds can provide some insight and support, their statistical power is limited by the relatively small number of compounds and high variability. However, by exploiting earlier work on this data set,^20^ the calculation of removal rates is not limited to identified analytes and, in fact, can be extended to the full suite of analytes. Cluster analysis was used previously to categorize all compounds based on distinct characteristics such as their atmospheric variability, chemical structure, reactivity, and origin, which provide context for interpreting removal rates for these compounds even in the absence of identification. All 408 analytes were previously categorized into eight clusters based on their temporal variations using the spherical k-means algorithm. The average carbon oxidation state of each analyte was estimated using chemical characterization by chromatography–mass spectrometry random forest modeling (Ch3MS-RF), a random forest model for characterization of unidentifiable compounds separated in GC/MS.^26^ The average carbon oxidation states and removal rates of all identified compounds are summarized in Table S1. Of these eight clusters, four were identified as instrument operational clusters (internal standards, contaminants, etc.), while the remaining four were classified as atmospheric clusters representing real-world data. While removal-rate variability is analyzed for each compound individually, examining all the compounds in a cluster as a whole provides broader interpretive insight into gases with certain characteristics. Removal rates of operational clusters do not provide insight into ambient atmospheric processes but support the conclusion that the method is not introducing bias with tight distributions centered around zero and statistically significantly different from zero, as expected for internal signals (Figure S3).

Most unidentified compounds in the atmospherically relevant clusters are observed to undergo reductions in concentration during precipitation events. In other words, most of these compounds have median removal rates statistically significantly higher than zero. Furthermore, three of the four clusters representing ambient analytes have median cluster removal rates statistically significantly higher than zero, indicating that the types of compounds represented by these clusters are removed during precipitation. Median removal rates for each cluster approximately align with observations for the representative identified compounds within each cluster (Figure 5): biomass burning (0.07 h^–1^), miscellaneous compounds including some biogenic oxidation products (0.03 h^–1^), terpenes (0.04 h^–1^), and isoprene oxidation compounds (0.09 h^–1^). Though the median removal rate of the compounds associated with terpenes is positive, the mean of the distribution is not statistically different from zero or from the distribution of the instrument operating clusters, in agreement with the conclusion that compounds that coexist with terpenes may be highly variable but, on average, are negligibly impacted by precipitation. Like the identified terpenes, compounds associated with this cluster may undergo complex biological processes that drive changes in the emissions during precipitation. For the other compound classes, concentrations significantly decrease during precipitation events.

**Figure 5:**
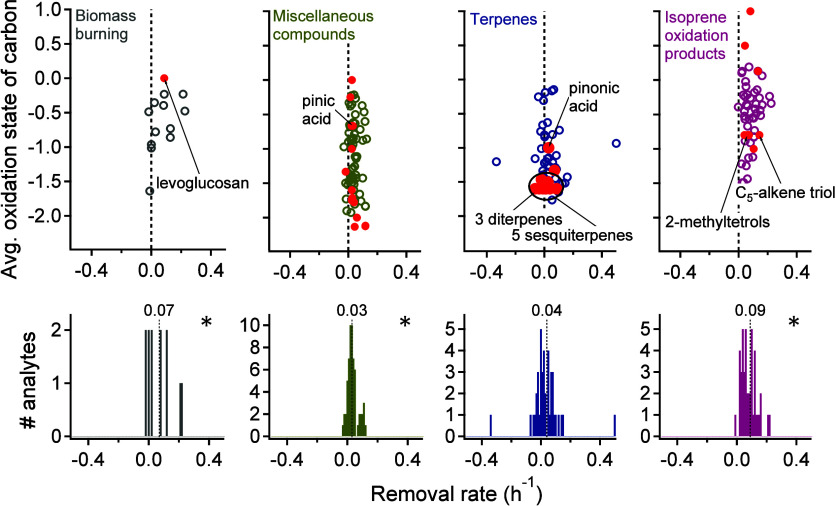
Median removal rates (scatter against oxidation state and histogram) of analytes in clusters identified as related to biomass burning, miscellaneous compounds, terpenes, and isoprene oxidation products. Average carbon oxidation states of analytes were estimated by Ch3MS-RF. Identified compounds are represented as red-filled circles within each cluster and are named Table S1. Labeled compounds are those included in Figure 4. Blue stars (*) on the upper right corner of histograms indicates that the removal rate mean of the cluster is statistically significantly different from the removal rate means of instrument operational clusters. A version of this figure including standard error for all analytes is available as Figure S4.

In general, observations of analytes within these clusters support the conclusions drawn from identified compounds but uniquely provide high statistical confidence given the large number of compounds. The clusters containing the most efficiently removed carbons are those associated with biomass burning tracers and isoprene oxidation products, but likely for different physicochemical reasons. The observed biomass burning compounds, including levoglucosan, are predominantly in the particle phase, so particle scavenging is their principal removal pathway despite the high solubility of any gas-phase component. Their strong polarity and hygroscopic character promote rapid water uptake and growth, making these particles especially susceptible to below-cloud scavenging.^41^ Field observations confirm efficient below-cloud removal of biomass burning aerosol during rainfall.^42^ In contrast, the isoprene oxidation products are largely in the gas phase, and their high Henry’s Law constants enable rapid dissolution into raindrops. Median removal rates of nearly all compounds in both of these clusters are positive, with the highest rate observed at 0.22 h^–1^. The representative identified compounds for each cluster shown in Figure 4 are near the median removal rate for each cluster. Like levoglucosan, many of the compounds in the biomass burning tracers cluster have a large particle fraction (ranging from 0.1 to 1.0, with a median of 0.82), suggesting that the observed removal rate is representative of that for particles, while the isoprene oxidation product cluster is representative of that for highly oxygenated gases. Analytes that exhibit the highest removal rates (e.g., 0.22 h^–1^) may provide the true upper bound for the efficiency of removal by wet deposition or may simply demonstrate inherent real-world variability in atmospheric concentrations and in characteristics of the precipitation. Notably, this fastest observed removal rate is in good agreement with the 4–6 h modeled time scale for wet scavenging of highly soluble gases at this location.

Compounds in the miscellaneous and terpene clusters provide some insight into the variability in removal during precipitation, with many compounds not exhibiting the clear trends toward removal observed for highly soluble gases. The miscellaneous cluster comprises analytes from various chemical groups, including alkanes and their oxidation products, α-pinene oxidation products, such as pinic acid, and directly emitted compounds, such as homosalate. The average diel pattern of this cluster exhibits high concentrations during daytime, aligning with the diel pattern of pinic acid.^20^ This suggests that some analytes within this cluster may be oxygenated products of biogenic organic compounds, potentially contributing to the increased average removal rate observed for this cluster and the subset of compounds observed to have high removal rates (although pinic acid tends to fall into the lower removal rate mode). Despite the overall diurnal trend in the diel pattern, rapid fluctuations in the concentration are also observed throughout the sampling period. Many identified analytes within this cluster also appear on the list of external standards, implying that residuals from previous samples may introduce noise, leading to their grouping in this cluster. The presence of both oxygenated compounds and external standards may account for the overall moderately low, but nonzero, average removal rate of compounds in this cluster. Though there are compounds associated with this cluster that have relatively high, statistically significant removal rates, it is difficult to characterize this process for any specific type or class of compounds, because there are no clear shared characteristics for compounds in this cluster. While these clusters include statistically significant removal of identified soluble gases and unidentified compounds, the variability of removal rates for compounds associated with the miscellaneous cluster suggests it is difficult to discern strong concentration changes during precipitation for intermediately soluble gases.

In the terpene cluster, low solubility classes show high variability and no consistent statistically significant removal. The average removal rate of analytes associated with the terpene cluster was 0.04 h^–1^, but the variability is large enough that the mean is statistically indistinguishable from zero. Although some of the compounds in the terpene cluster overlap in oxidation state of carbon with compounds in the miscellaneous and biomass burning clusters, terpenoids are generally nonpolar hydrocarbons that stay mostly in the gas phase. Their low solubility in water makes dissolution into raindrops inefficient, so it makes sense that the median removal rate of terpene-associated compounds is indistinguishable from zero. Collectively, these two clusters demonstrate that while high solubility gases demonstrate a clear trend toward removal, other gases have more variability with changes during precipitation that are difficult to quantify, parametrize, or even definitively classify as a decrease.

## 4 Atmospheric Implications

The cluster that contains isoprene oxidation products is particularly valuable for evaluating rain-induced concentration change. These observations are some of the first direct constraints on not only the net effect of rain on these gases (wet scavenging together with any co-occurring processes) but also the degree to which our models and understanding of below-cloud scavenging reflect real-world conditions. This cluster contains 44 analytes that are primarily gas phase and are expected to be highly oxidized, providing a statistically robust examination of the removal of soluble gases during precipitation. The average removal rate is on the order of 10% per hour, and removal rates for these gases range from −0.003 to 0.22 h^–1^, with the second smallest value being 0.02 h^–1^. Precipitation can therefore be assumed to be associated with a statistically significant net decrease of concentration in nearly all such relatively oxidized gases and to occur on effective time scales of as short as a few hours. Similar removal rates are observed for many compounds that are primarily in the particle phase, such as levoglucosan, but are not observed for hydrocarbons.

Consequently, while all the meteorological and atmospheric processes cannot be deconvoluted that may contribute to the observed reduction in concentration during rain events (wet deposition, advection, changes in other sources or sinks), observed reductions in concentration are strongly statistically significant and indicate real changes in concentrations during precipitation that can reasonably interpreted as being due at least in large part to below-cloud scavenging. Critically, the observed rates of removal for soluble gases are quantitatively consistent with model predictions. Bi and Isaacman-VanWertz give wet deposition time scale 4–6 h, corresponding to first-order rate constants of 0.25–0.17 h^–1^ for highly soluble gases, whereas the isoprene oxidation products cluster spans from 0.003 to 0.22 h^–1^ with a median of 0.09 h^–1^, an upper quartile of 0.14 h^–1^, and a maximum of 0.22 h^–1^, placing the fastest observations within the modeled rate for the most soluble gases. This work consequently not only provides critical observational constraints on removal during precipitation events, of which there are currently very few, but also suggests that our models built on first principles are reasonably descriptive of real-world processes.

Work remains to examine the extent to which the observed removal rates of particles align with the current models. Additionally, the large natural variability of the atmosphere limits the quantitative understanding of removal during precipitation for less soluble gases. For example, precipitation intensity is expected to be directly correlated to the rainfall rate. In this work, a strong quantitative relationship between removal rate and intensity is not observed (Figure S7), though the highest intensity precipitation events more strongly exhibit a clear tendency to remove oxygenated gases such as the isoprene oxidation products. This result qualitatively supports previous modeling work suggesting that rain frequency and duration are more important drivers than precipitation droplet size distribution or intensity.^10^ Similarly, low-intensity precipitation events do not have a clear tendency to remove even soluble gases, consistent with previous work on particles that found very low scavenging rates during rain intensities below ∼1 mm/h.^11^ The inability to discern parametrizable quantitative relationships is likely due to differences in analyte properties, inherent variability, and complexity of the atmosphere, and potential convolution with co-occurring processes such as entrainment from cleaner higher altitudes. However, decreases during precipitation are not consistently observed for less soluble gases, suggesting that a Henry’s law-driven process, such as below-cloud scavenging, is a major contributor to the observed decreases of soluble gases during precipitation events. Overall, our observations indicate that net decreases during precipitation for highly water-soluble gases occur at rates of ∼10% per hour, consistent with models that predict efficient below-cloud scavenging approaching the irreversible-uptake limit for such gases.

## Supplementary Material





## Notes

Compound-specific data: average carbon oxidation state and removal rate of identified compounds (XLSX)
Additional precipitation data, statistical results, and a list of removal rates for identified compounds (PDF)
