# Supplementary material for: Impacts of Precipitation Events on Concentrations of Oxygenated Gas- and Particle-Phase Compounds Observed in the Amazon
Source: ACS EST Air. 2025 Nov 6;2(11):2422–32. doi: 10.1021/acsestair.5c00163 (PMC12624733; doi:10.1021/acsestair.5c00163)
Supplement: Supplementary file 2 [file ea5c00163_si_002.pdf]

Supplementary Information for:

Impacts of precipitation events on concentrations of oxygenated gas- and particle-phase compounds observed in the Amazon

*Sungwoo Kim and Gabriel Isaacman-VanWertz**

Charles E. Via Jr. Department of Civil and Environmental Engineering, Virginia Tech, Blacksburg, VA 24061, USA

***Corresponding author.** Gabriel Isaacman-VanWertz: ivw@vt.edu

S1. Rainfall and wind speed overview.

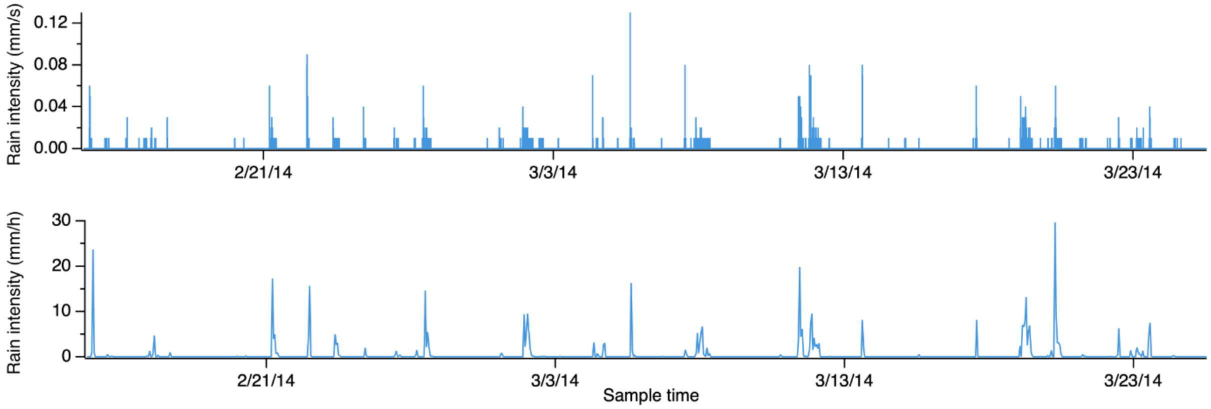

Figure S1. Precipitation amounts at site T3 from February 14 to March 25, 2014, with data recorded every second (a) and aggregated into hourly totals (b).

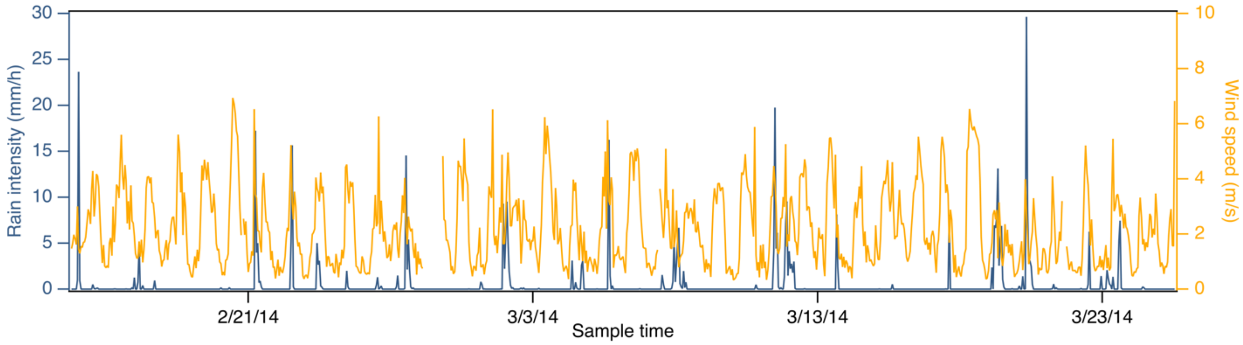

Figure S2. The rainfall intensity (blue) and wind speed (yellow) recorded during the sampling period.

S2. Instrument operational clusters.

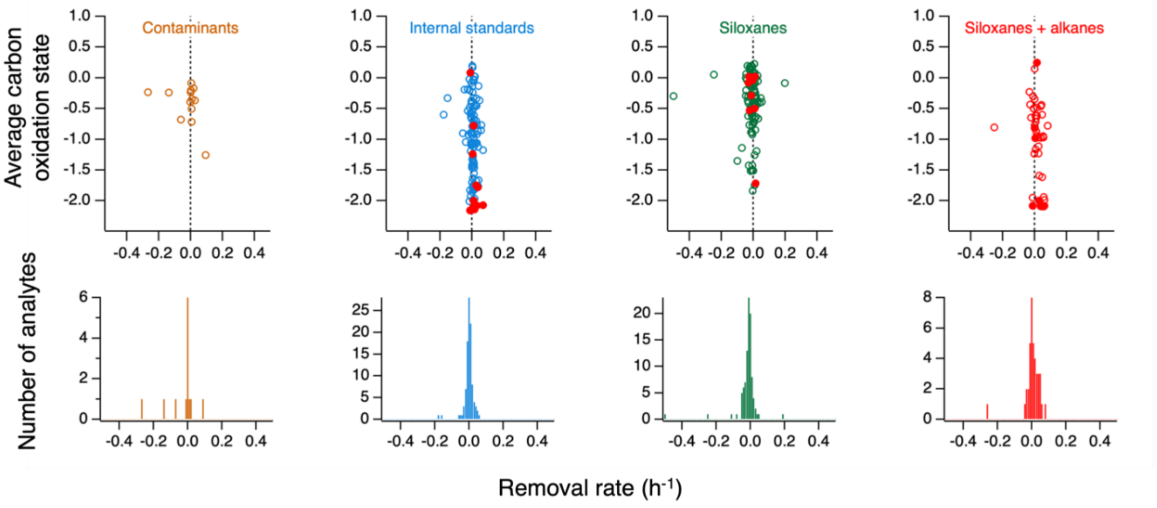

Figure S3. Median removal rates (scatter against oxidation state and histogram) of analytes in instrument operational clusters (contaminants, internal standards, siloxanes, and siloxane alkane mixture). Average carbon oxidation states of analytes were estimated by Ch3MS-RF. Identified compounds are represented as red-filled circles within each cluster and are named in Table S1.

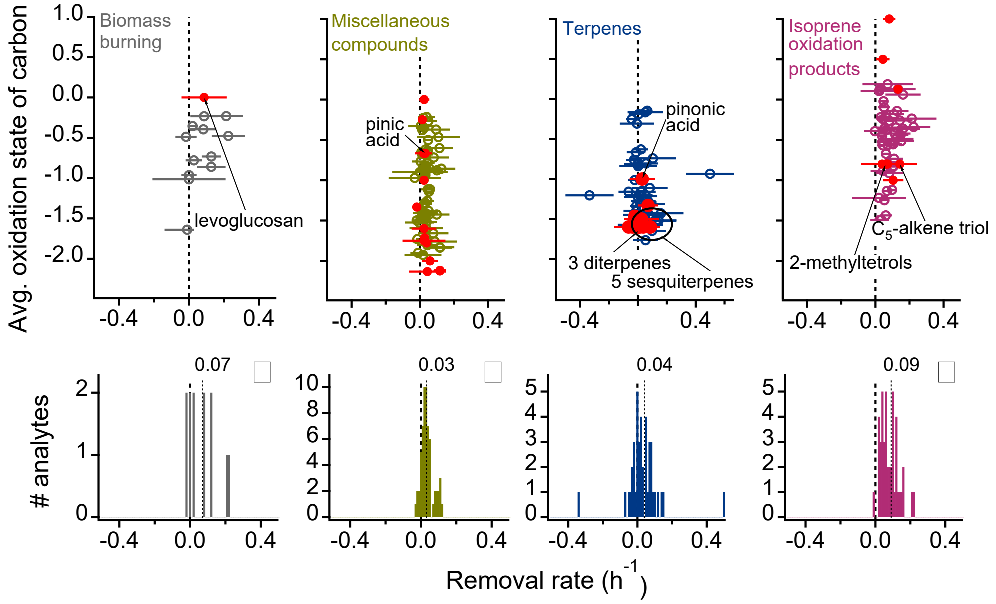

Figure S4. Median removal rates (scatter against oxidation state and histogram) of analytes in clusters identified as related to biomass burning, miscellaneous compounds, terpenes, and isoprene oxidation products. Average carbon oxidation states of analytes were estimated by Ch3MS-RF. Identified compounds are represented as red-filled circles within each cluster and are named in Table S1. Labeled compounds are those included in Figure 4. Error bars are standard error of each analyte. Blue stars (*) on the upper right corner of histograms represent the true removal rate means of the clusters are statistically significantly different from the removal rate means of instrument operational clusters.

S3. Statistical significance of rain events.

To determine whether large positive removal rates can be statistical artifacts, the removal rates during rain events of all analytes in the isoprene oxidation product cluster were compared to the calculated removal rate (i.e., fractional change in concentration) during periods without precipitation. The average fractional change in concentration for all analytes in the isoprene oxidation products cluster for non-precipitation periods was clustered around 0 with an average of 0.00 h^{-1} and a standard deviation of 0.06 h^{-1} (Figure S5), placing the removal rate of 0.09 h^{-1} for this cluster well outside the range of one standard deviation. An intuitive understanding of this statistical significance is provided by comparing the median removal rate for all analytes in the isoprene oxidation product cluster during the 73 precipitation datapoints to randomly selected sets of 73 datapoints as shown in Figure S6, and precipitation is found to yield substantially higher changes in concentration.

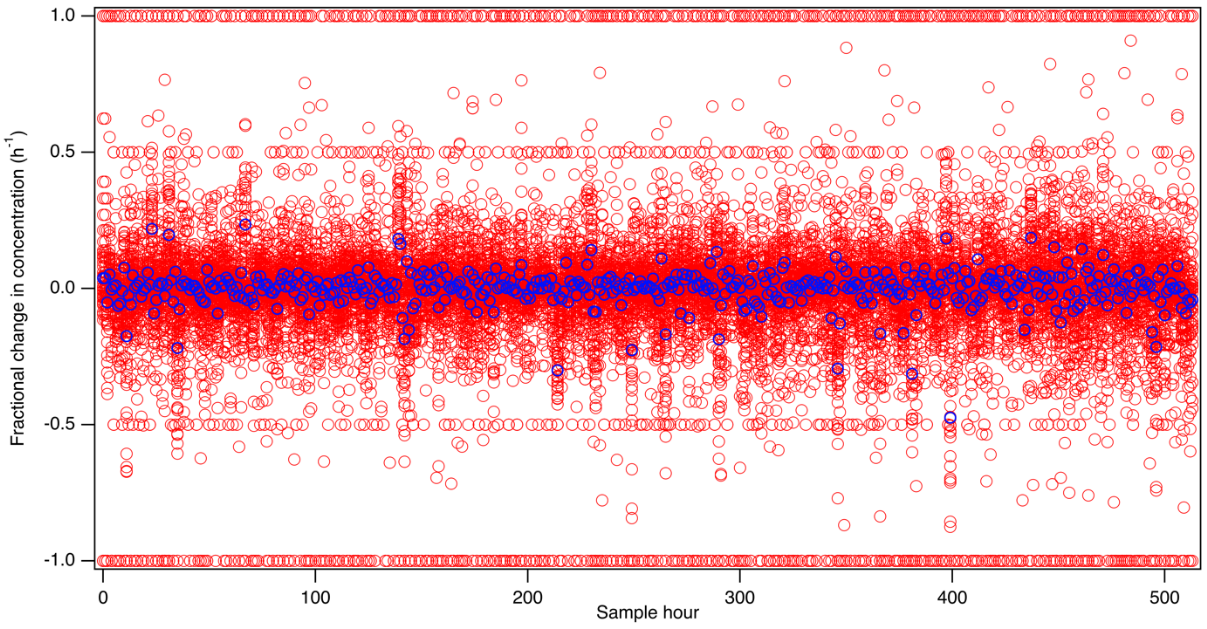

Figure S5. Fractional concentration change values of isoprene oxidation products estimated during periods of zero rainfall (red circles) overlaid with the median values at each sample hour (blue circles).

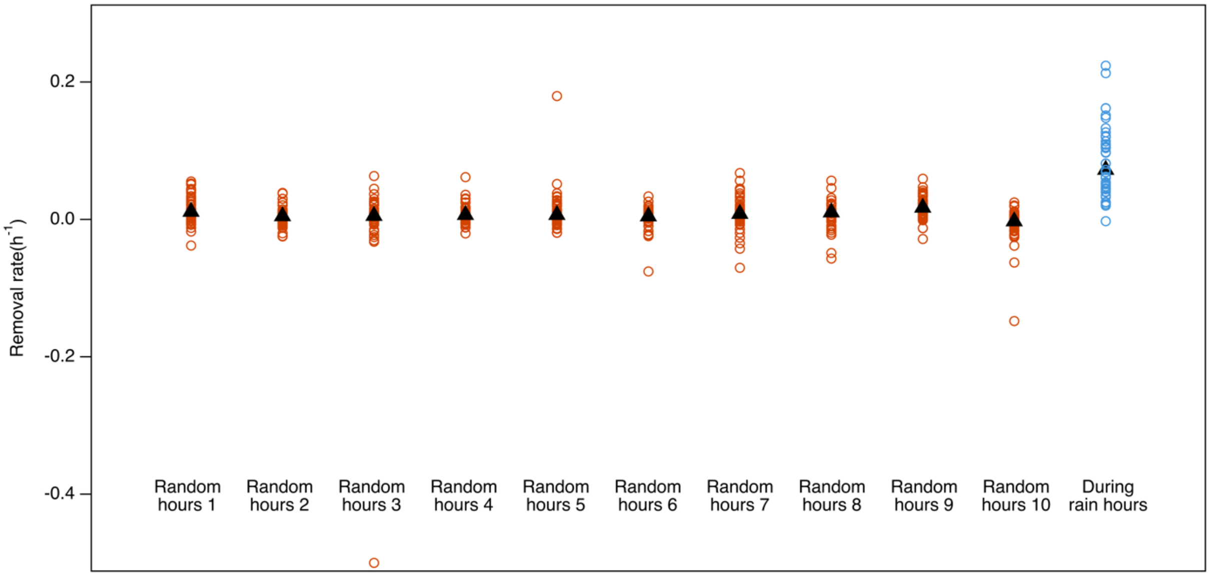

Figure S6. Removal rates (or fractional change in concentration for test sets) of all analytes in the isoprene oxidation products cluster. Each empty circle represents the median removal rate (i.e., fractional change) of a single analyte within the isoprene oxidation product cluster for 73 datapoints. The 73 datapoints for which precipitation rate is greater than 1 mm/h are shown in blue, with randomly selected sets in red. The median value of each test dataset is depicted as a black triangle.

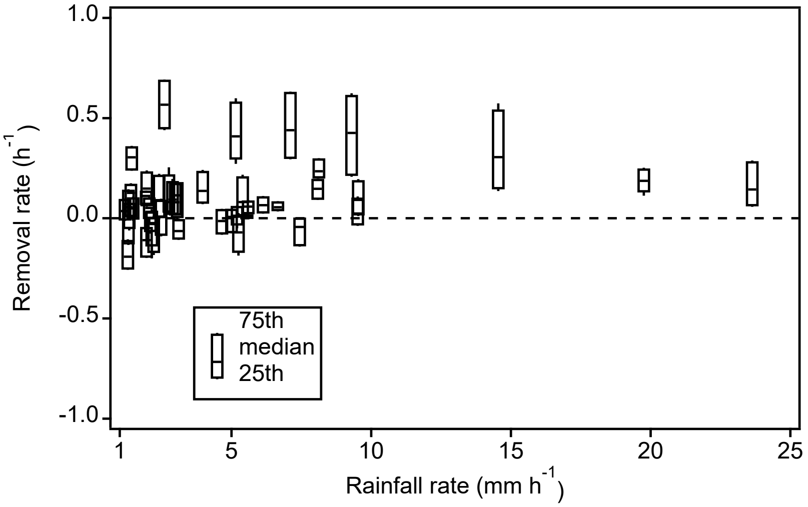

Figure S7. Removal rate of the cluster of isoprene oxidation products as a function of rainfall intensity. Box and whiskers show median and interquartile range of all analytes in the cluster for a given rainfall event. Only events with intensity > 1 mm h^{-1} are shown.

S4. Assessment of downdraft mixing via ozone rain comparison

To test whether convective downdrafts systematically perturb near-surface composition during our rain events, we examined ozone (O_3) behavior around the start of precipitation. The 1 s rain record was aggregated to 1 min totals (mm/min). A rain onset was defined as the first minute with >0 mm/min following a dry minute (0 mm/min). Only nighttime events (20:00–05:00 Local Time) were analyzed. For each onset we extracted a ±120 min window centered on the first rainy minute ($t = 0$). Ozone was normalized by the mean O_3 during the first 30 min of that window (−120 to −90 min). Minute-by-minute normalized values were then averaged across all qualifying events to produce a composite trace (Figure S7).

The composite shows no systematic increase in O_3 during or after precipitation; the curve remains within a few percent of unity throughout the window, indicating no obvious, repeatable downdraft signature in near-surface ozone during these nighttime rains. These results do not eliminate all rain-induced mixing or prove downdrafts were absent. A more detailed meteorological analysis would be required to fully confirm the magnitude and the impact of downdrafts. Nevertheless, the lack of a systematic ozone response gives reasonable confidence that downdrafts are not the dominant driver of the rain-period concentration changes.

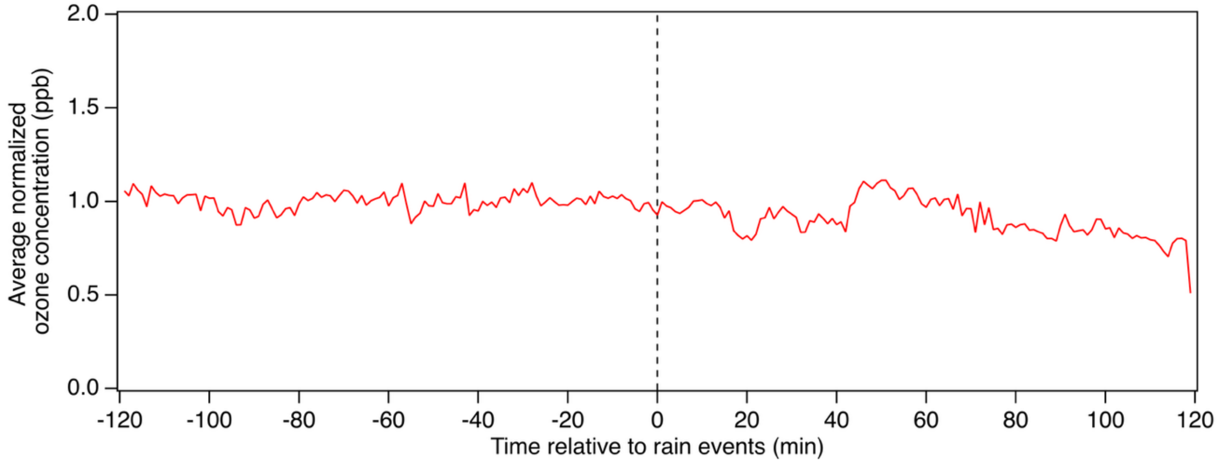

Figure S8. Normalized ozone around nighttime (20:00 – 5:00 LT) rain onsets. Red line shows the mean ozone concentration for all events, with $t = 0$ marking the first rainy minute. A value of 1 denotes no change relative to the pre-event baseline.

S5. Instrumental and statistical uncertainty of removal rates.

Individual removal rates are ratios of two concentration measurements, which has been formally and empirically shown for this instrument to yield uncertainty of 15% (Isaacman et al., 2014). Specifically, however, this percentage is uncertainty in the ratio, not the removal rate – at a ratio of 1 (i.e., samples before and after rain on the same), the removal rate is nominally 0 h^{-1} while the uncertainty suggests a ratio between 0.85 or 1.15, equal to a removal rate as high as 0.15-0.2 h^{-1}; uncertainty decreases as the ratio decreases and removal rate increases (e.g., there is low uncertainty when there is zero signal in C_t). Formally, then, uncertainty in the measurement of each hourly removal estimate is in the range of 0.15 h^{-1} for low removal rates down to 0.015 h^{-1} when the removal rate is 0.9 h^{-1}, and can be calculated as: $\boldsymbol{\sigma} = 0.15 - 0.15R$, where R is the calculated removal rate. These estimates do not capture scatter caused by real-world fluctuations in concentrations that are due to changing atmospheric processes. Due to the inherent variability of the atmosphere, the overall impact of precipitation on concentrations can be assessed from the central tendencies of larger numbers of datapoints rather than individual measurements, as demonstrated in detail by Laakso and co-workers (2003). Consequently, removal rates of one compound are best evaluated as the mean or median of all 43 rain events considered. The median removal rate of a single compounds can be known with a standard error (SE) estimated as:

$$SE_{compound} \approx 1.25\frac{\sigma}{\sqrt{n}},$$

where σ may be the calculated uncertainty of the median removal rate or may be the empirical standard deviation of the n number of observations (e.g., $n = 43$ rain events). The latter is more conservative as it captures uncertainty due to real-world variability and is used in this work.

The statistical strength of this work can be further increased by calculating the median removal rate of a cluster of similar compounds, which combines 43 rain events times the number of compounds in the cluster. In this case, the median rate can be known with a standard error that can be calculated using the same equation, using $n = m$ and $\boldsymbol{\sigma}$ = the standard deviation of the cluster.

S6. Compound specific data: average carbon oxidation state and removal rate of identified compounds.

Table S1. Average carbon oxidation states and removal rates of all 74 identified compounds, represented as red filled circles in Figures 4, 5, S3, and S4. The full dataset comprises 408 analytes. The remaining 334 compounds could not be definitively identified by spectral matching. Compound groups shown in Figure 4-5 are indicated by *, **, ***, ****, and ***** to represent internal standards, resolved terpenes, α-pinene oxidation products, biomass burning tracer, and isoprene oxidation tracers, respectively. The standard error (SE) for each compound's removal rate was computed from the empirical standard deviation of that compound's observations across all rain events.

Cluster	Compound	$\overline{OS_C}$	Removal rate (h^{-1})	SE (h^{-1})
Cluster 1	Levoglucosan, TMS ****	0.00	0.09	0.08
Cluster 3	Trisiloxane, 1,1,1,5,5,5-hexamethyl-3,3-bis[(trimethylsilyl)oxy]-	0.09	-0.01	0.02
	Silane, trimethyl(1-methyl-1-phenylethoxy)-	-0.78	0.01	0.03
	Dodecane, 3-methyl-	-2.15	-0.01	0.05
	d-C13	-2.15	-0.01	0.04
	C13	-2.15	-0.01	0.02
	2-(2-Butoxyethoxy)ethoxy-trimethylsilane	-1.24	0.00	0.02
	Tridecane, 5-methyl-	-2.14	0.00	0.08
	2,3-Dimethyldodecane	-2.14	0.01	0.02
	Tridecane, 3-methyl-	-2.14	0.01	0.03
	C14	-2.14	0.02	0.03
	d-C16 *	-2.13	0.01	0.01
	d-C18 *	-2.11	0.01	0.01
	d-C15 alcohol TMS *	-2.00	0.01	0.01
	d-C20 *	-2.10	0.01	0.01
	d-C16 acid TMS *	-1.75	0.03	0.01

	d-C22 *	-2.09	0.01	0.05
	d-C18 acid TMS *	-1.78	0.04	0.05
	d-C24 *	-2.08	0.02	0.09
	d-C26 *	-2.08	0.03	0.09
	d-C28 *	-2.07	0.01	0.16
Cluster 4	Butanoic acid, 4-[(trimethylsilyl)oxy]-, trimethylsilyl ester	-0.50	0.01	0.05
	Pentasiloxane, dodecamethyle-	-0.08	-0.03	0.04
	Benzoic acid, TMS	-0.29	-0.01	0.02
	Hexasiloxane, tetradecamethyl-	-0.03	-0.01	0.02
	Cyclohexasiloxane, dodecamethyl-	-0.53	-0.02	0.03
	Hexasiloxane, tetradecamethyl-	0.02	-0.03	0.01
	Cycloheptasiloxane, tetradecamethyl-	-0.04	-0.01	0.05
	d-pentaerythritol TMS	0.02	0.01	0.01
	d-C14 acid TMS	-1.71	0.01	0.01
Cluster 5	Glyoxime, bis(trimethylsilyl)-	0.00	0.03	0.03
	glycerol tris-TMS	-0.67	0.02	0.01
	C9 acid, TMS	-1.33	-0.02	0.02
	Benzoic acid, 2-[(trimethylsilyl)oxy]-, methyl ester	-0.25	0.02	0.02
	C10 Acid, TMS	-1.60	0.02	0.02
	C15	-2.13	0.04	0.06
	C16	-2.13	0.12	0.08
	pinic acid, TMS ***	-0.67	0.03	0.02
	C17	-2.12	0.12	0.07

	C14 acid, TMS	-1.71	0.03	0.02
	Hexadecanol, TMS	-2.00	0.06	0.02
	C15 acid, TMS	-1.73	0.03	0.02
	Homosalate, TMS	-1.00	0.02	0.05
	C16 Acid, TMS	-1.75	0.02	0.02
	C18 Acid, TMS	-1.78	0.04	0.06
Cluster 6	C8 Acid, TMS	-1.50	0.03	0.02
	Copaene **	-1.60	0.01	0.04
	α-Gurjune'ne **	-1.60	0.01	0.07
	α-patchoulene **	-1.60	-0.06	0.07
	UNK_SESQ	-1.60	0.04	0.09
	SESQ_O **	-1.60	-0.04	0.10
	trans-Calamenene **	-1.47	-0.02	0.05
	pinonic acid ***	-1.00	0.03	0.03
	UNK_DITERP **	-1.33	0.07	0.04
	UNK_DITERP **	-1.60	0.04	0.09
	DITERP_C **	-1.60	-0.01	0.04
	DITERP_D	-1.60	0.03	0.12
Cluster 7	Silanol, trimethyl-, phosphate (3:1)	-0.82	0.00	0.05
	1,4-Benzenedicarboxylic acid, bis(trimethylsilyl) ester	0.25	0.01	0.05
	C21	-2.10	0.05	0.06
	Octadecanol, TMS	-2.00	0.03	0.05
	C22	-2.09	0.06	0.05

	C23	-2.09	0.04	0.09
	Cyclotrisiloxane, 2,4,6-trimethyl-2,4,6-triphenyl-	-0.98	0.00	0.09
	C24	-2.08	-0.01	0.10
	C25	-2.08	0.06	0.11
Cluster 8	Cyclohexasiloxane, dodecamethyl-	0.13	0.13	0.10
	1,2,3-butanol isomer	-1.00	0.10	0.05
	Butanedioic acid, bis(trimethylsilyl) ester	0.50	0.04	0.03
	2-Butenedioic acid (E)-, bis(trimethylsilyl) ester	1.00	0.08	0.03
	C5 alkene triol 3 *****	-0.80	0.14	0.05
	C5 alkene triol 1	-0.80	0.07	0.15
	methyl tetrol 2 *****	-0.80	0.07	0.03
	methyl tetrol 1 *****	-0.80	0.08	0.04

S7. Rain duration statistics.

Table S2. Mean rainfall duration for hourly precipitation events, binned by intensity. “Count” is the number of wet hours in each bin. “Mean (s)” and “Mean (min)” give the average total rain duration per hour, expressed in seconds and minutes, respectively.

Bin (mm/h)	Count	Mean (s)	Mean (min)
1-2	16	144.31	2.41
2-5	25	285.00	4.75
5-10	24	600.17	10.00
>10	7	1248.57	20.81

S8. Estimated Henry's law constants for resolved terpenes and their relation to removal rates.

Table S3. Estimated Henry's law constants for the four identified terpenes. Values were obtained with US EPA EPI suite HENRYWIN module. The table also lists each compound's solubility in water (mg/L) and median removal rate (h^{-1}).

Compound	Henry's law constant		Water solubility (mg/L)		Removal rate (h^{-1})
	(atm-m3/mol)	(M/atm)	WS from Kow	WaterNT	
Copaene	0.19	5.30E-03	0.32	0.03	0.012
α-Gurjunene	0.23	4.30E-03	0.06	0.05	0.006
α-patchoulene	0.19	5.30E-03	0.15	0.04	-0.064
trans-Calamenene	8.29E-03	1.20E-01	0.2	0.25	-0.020

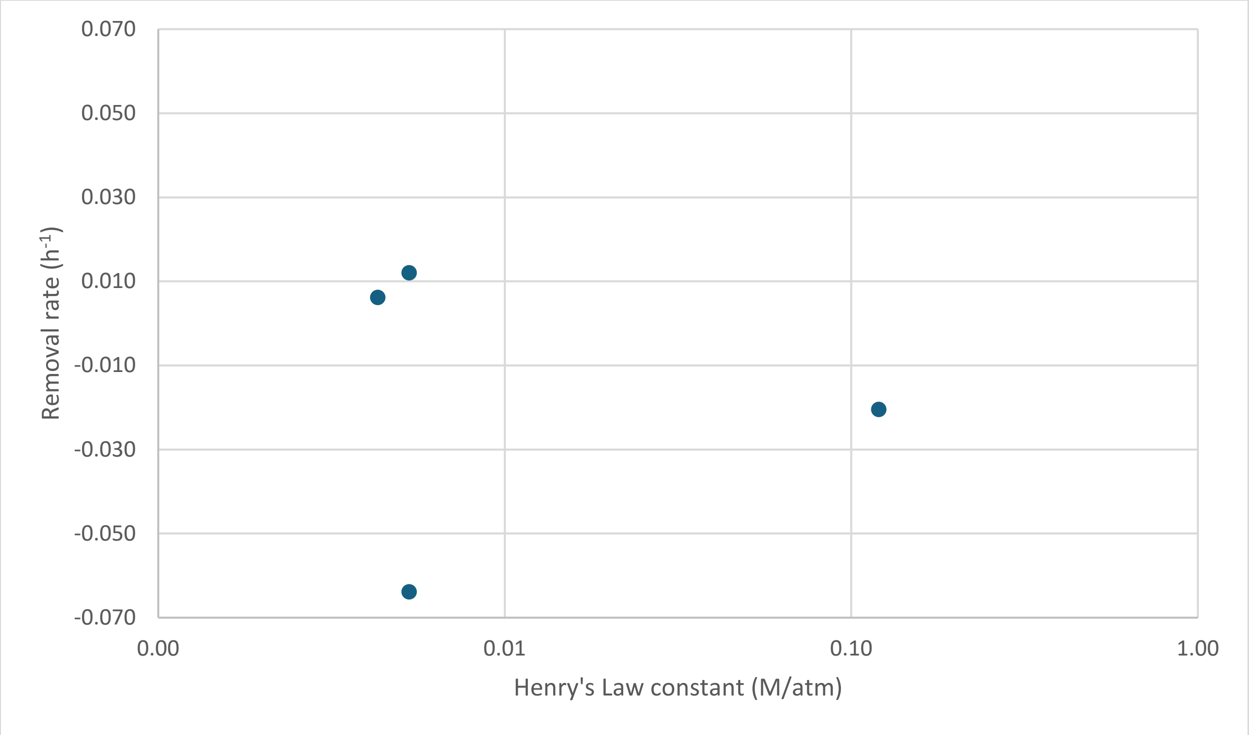

Figure S9. Relationship between terpene solubility and removal efficiency.
